# Interspecies Interactions in Relation to Root Distribution Across the Rooting Profile in Wheat-Maize Intercropping Under Different Plant Densities

**DOI:** 10.3389/fpls.2018.00483

**Published:** 2018-04-20

**Authors:** Yifan Wang, Yazhou Qin, Qiang Chai, Fuxue Feng, Cai Zhao, Aizhong Yu

**Affiliations:** ^1^Gansu Provincial Key Laboratory of Arid Land Crop Science, Lanzhou, China; ^2^College of Agronomy, Gansu Agricultural University, Lanzhou, China; ^3^Agricultural Shaya County Service, Xinjiang, China; ^4^School of Engineering, Gansu Agricultural University, Lanzhou, China

**Keywords:** intercropping, interspecies interaction, plant density, root weight density, root length density, root surface area density, recovery effect, grain yield

## Abstract

In wheat-maize intercropping systems, the maize is often disadvantageous over the wheat during the co-growth period. It is unknown whether the impaired growth of maize can be recovered through the enhancement of the belowground interspecies interactions. In this study, we (i) determined the mechanism of the belowground interaction in relation to root growth and distribution under different maize plant densities, and (ii) quantified the “recovery effect” of maize after wheat harvest. The three-year (2014–2016) field experiment was conducted at the Oasis Agriculture Research Station of Gansu Agricultural University, Wuwei, Northwest China. Root weight density (RWD), root length density (RLD), and root surface area density (RSAD), were measured in single-cropped maize (M), single-cropped wheat (W), and three intercropping systems (i) wheat-maize intercropping with no root barrier (i.e., complete belowground interaction, IC), (ii) nylon mesh root barrier (partial belowground interaction, IC-PRI), and (iii) plastic sheet root barrier (no belowground interaction, IC-NRI). The intercropped maize was planted at low (45,000 plants ha^−1^) and high (52,000 plants ha^−1^) densities. During the wheat/maize co-growth period, the IC treatment increased the RWD, RLD, and RSAD of the intercropped wheat in the 20–100 cm soil depth compared to the IC-PRI and IC-NRI systems; intercropped maize had 53% lower RWD, 81% lower RLD, and 70% lower RSAD than single-cropped maize. After wheat harvest, the intercropped maize recovered the growth with the increase of RWD by 40%, RLD by 44% and RSAD by 11%, compared to the single-cropped maize. Comparisons among the three intercropping systems revealed that the “recovery effect” of the intercropped maize was attributable to complete belowground interspecies interaction by 143%, the compensational effect due to root overlap by 35%, and the compensational effect due to water and nutrient exchange (CWN) by 80%. The higher maize plant density provided a greater recovery effect due to increased RWD and RLD. Higher maize plant density stimulated greater belowground interspecies interaction that promoted root growth and development, strengthened the recovery effect, and increased crop productivity.

## Introduction

Intercropping refers to a planting pattern where two or more crops are grown in alternate rows in the same field (Vandermeer, 1989). The intercropping systems have been proved to be superior to single cropping in productivity, because they promote a higher land utilization efficiency (Romero et al., 2013), optimize the use of available resources in both time and space (Fan et al., 2013; Yin et al., 2015), and reduce weed and disease pressures (Agegnehu et al., 2008). The yield advantage in intercropping systems is often obtained through the coordination of the interspecies interaction for above- and/or belowground competition (Li et al., 2006). Belowground competition often takes various forms and involves complex processes (Schenk, 2006), where the intercrops may compete for available water and nutrients during the co-growth period, leading to poor performance of one crop over the other one (Wilson, 1988). However, well-coordinated interspecies interaction may result in positive outcomes due to improved resource sharing and temporal optimization for the growth of the aboveground plant parts. Therefore, understanding the interspecies interactions belowground may provide a guideline for a better coordination of the potential competition for resources for above ground plant parts.

Plant roots serve as the crucial site for belowground interspecies interactions, because roots not only absorb water and nutrients, but also synthesize and transform other trace substances to the other plant tissues (Landhäusser et al., 2006; Hu et al., 2009). The growth of crop roots is related to many crop management factors, such as irrigation and fertilizer management (Levangbrilz and Biondini, 2003; Ahmad et al., 2010), spatial arrangement (Wang et al., 2006; Yang et al., 2010), and plant density (Muoneke et al., 2007). Often the case that intercropped plants have higher root mass than monoculture crops (Li et al., 2006), because the overlapping of the roots of the two intercropped crops makes full use of belowground resources (Vandermeer, 1989); this belowground ecological niche separation plays a key role in determining the growth and yield of the intercrops (Li L. et al., 2011). Efforts to optimize the root growth may help regulate the interspecific relationship in the intercropping system (Li et al., 2006). We proposed that manipulation of planting densities could promote a positive interspecific interaction in intercropping systems because the root mass of the intercrops is often a reflection of plant density when growth resources are sufficiently available. However, little information is available in regard to how the planting density of one intercrop may influence the root dynamics and the function of the other intercrop. Some studies have shown that increasing the density of one of the intercrops increased interspecific competition and reduced the yield of the other component crop (Beaver and Melgar, 1999; Muoneke et al., 2007). However, it is unknown if this kind of competition may occur under irrigation where competition for soil water is minimal.

Wheat/maize intercropping has been widely adopted in the primary grain production areas of Northwestern China, where the cropping system has traditionally been a single crop annually due to temperature constraints (Qin et al., 2013). Research has shown that in the intercropping system, early-planted wheat has a competitive advantage over late-planted maize (Hu et al., 2016). Although the intercropped maize initially suffers growth penalties during the co-growth period due to late-planting, the maize continues to grow, after wheat harvest, until the season end with higher above- and belowground growth rates compared with single-cropped maize. In a normal growing season, intercropped maize can have a “recovery effect” (Yin et al., 2017), which is manifested in an increase in aboveground dry matter accumulation. However, it is not clear how belowground interspecies interactions may affect the recovery effect. In efforts to elucidate the mechanisms underlying the relationship between belowground interspecies interaction and the recovery effect, we used a root partitioning technique, as discussed by others (Ong, 1995). We designed three types of root barrier treatments to investigate belowground interactions: (i) no root barrier (allowing a complete belowground interaction), a nylon mesh barrier (allowing a partial belowground interaction), and a plastic sheet barrier (no belowground interspecies interaction). The intercropped maize was planted at two plant densities (45,000 and 52,000 plants ha^−1^). The objective of this study were to determine (i) the vertical distribution characteristics of roots in responses to different belowground interactions, (ii) the recovery effect of intercropped maize roots after wheat harvest, and (iii) the relationships between root vertical distribution and recovery effect in affecting grain yield. Our hypothesis was that complete belowground interaction under high maize density promotes root growth of the intercropped wheat and maize, strengthens the root recovery effect of maize at wheat postharvest, and thereby improving intercropping productivity.

## Materials and methods

### Experimental site

**Figure 1:**
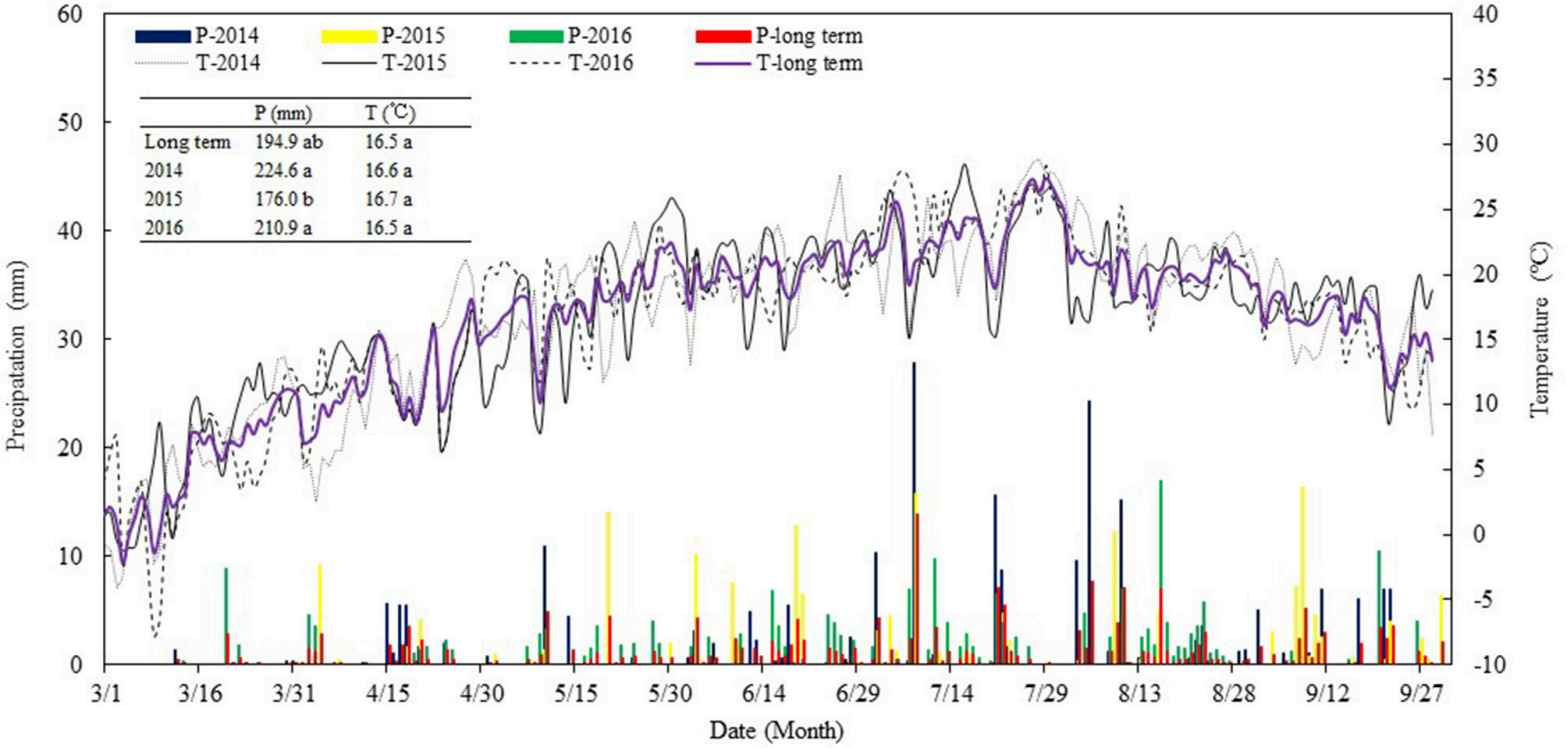
Precipitation (P) and air temperature (T) during maize growing seasons (from 1st Mar. to 30th Sept.) in 2014, 2015, and 2016 in comparison with the long-term (from 2000 to 2017, and getting the data from the National Meteorological Information Center) averages, at Wuwei Experimental Station.

Field experiments were carried out from 2014 to 2016 at the Wuwei Oasis Experimental Station (37°96′N, 102°64′E, elev. 1,506 m.) of Gansu Agricultural University, located in the eastern region of the Hexi Corridor in Northwest China. The long-term (1960~2009) annual sunshine duration is 2,945 h, annual mean air temperature is 7.2°C, and a frost-free period is 155 days (Chai et al., 2014). The meteorological records from the National Meteorological Information Administration of China (Beijing, China) show that the long-term mean annual precipitation is 156 mm with 70% occurring during the May-September growing season, while annual evaporation (free-water surface) is >2,400 mm. In the present study, we recorded some relevant weather data using a Farmland Microclimate Automatic Monitoring System (Hangzhou, China). Sunshine duration during the study years of 2014, 2015, and 2016 was 3,004, 2,837, and 2,947 h, respectively; average air temperature was 6.3, 7.9, and 7.0°C; frost-free period was 162, 123, and 158 days; and annual precipitation was 245, 167, and 210 mm, respectively (Figure 1). The precipitation during the study years was higher than the long-term averages, and this factor was considered when we determined the total amount of irrigation water applied to the crops. Temperatures in each of the study years were comparably similar to the long-term averages. Overall, the abundant sunshine and temperatures in the area are more than the need by a crop per year but are insufficient for the needs of two crops per year. Intercropping of a cool-season, earlier-maturing crop with a warm-season, later-maturing crop is ideal to enhance the use of the natural resources.

### Experimental design and plot management

The experiment included nine treatments that were arranged in a randomized complete block design with three replicates. Three root partitioning patterns for the wheat-maize intercropping were performed at two maize plant densities, i.e., no root barrier, a nylon-mesh barrier, and a plastic sheet barrier, each at the low (45,000 plants ha^−1^) and high (52,500 plants ha^−1^) plant densities. Corresponding monoculture treatments included single-cropping maize at the plant densities of 90,000 and 105,000 plants ha^−1^, and single-cropping wheat at the density of 6,750,000 plants ha^−1^. The intercropped wheat was at 3,750,000 plants ha^−1^. The maize plant densities were ensured using an in-house maize planter where the distance between plants within a row was justified according to the density. The wheat density was ensured with a rate of viable seed per area based on seed size, germination rate, and field emergence rate. To simplify the presentation, we designated the wheat-maize intercropping with no root barrier at the low and high plant densities as IC1 and IC2 where “IC” representing “Intercropping” and the numbers followed as the low (1) and high (2) maize plant densities, respectively; thus, the wheat-maize intercropping with nylon-mesh root barrier (Partial root interaction) at the low and high densities was designated as IC-PRI1 and IC-PRI2; and the wheat-maize intercropping with a plastic sheet barrier (No belowground interaction) at the low and high densities as IC-NRI1 and IC-NRI2; single maize cropping at the low and high densities as M1 and M2, and single wheat cropping as W. The plant densities used in the experiment were based on the results of previous studies conducted at the area (Li et al., 2001b; Hu et al., 2014).

**Figure 2:**
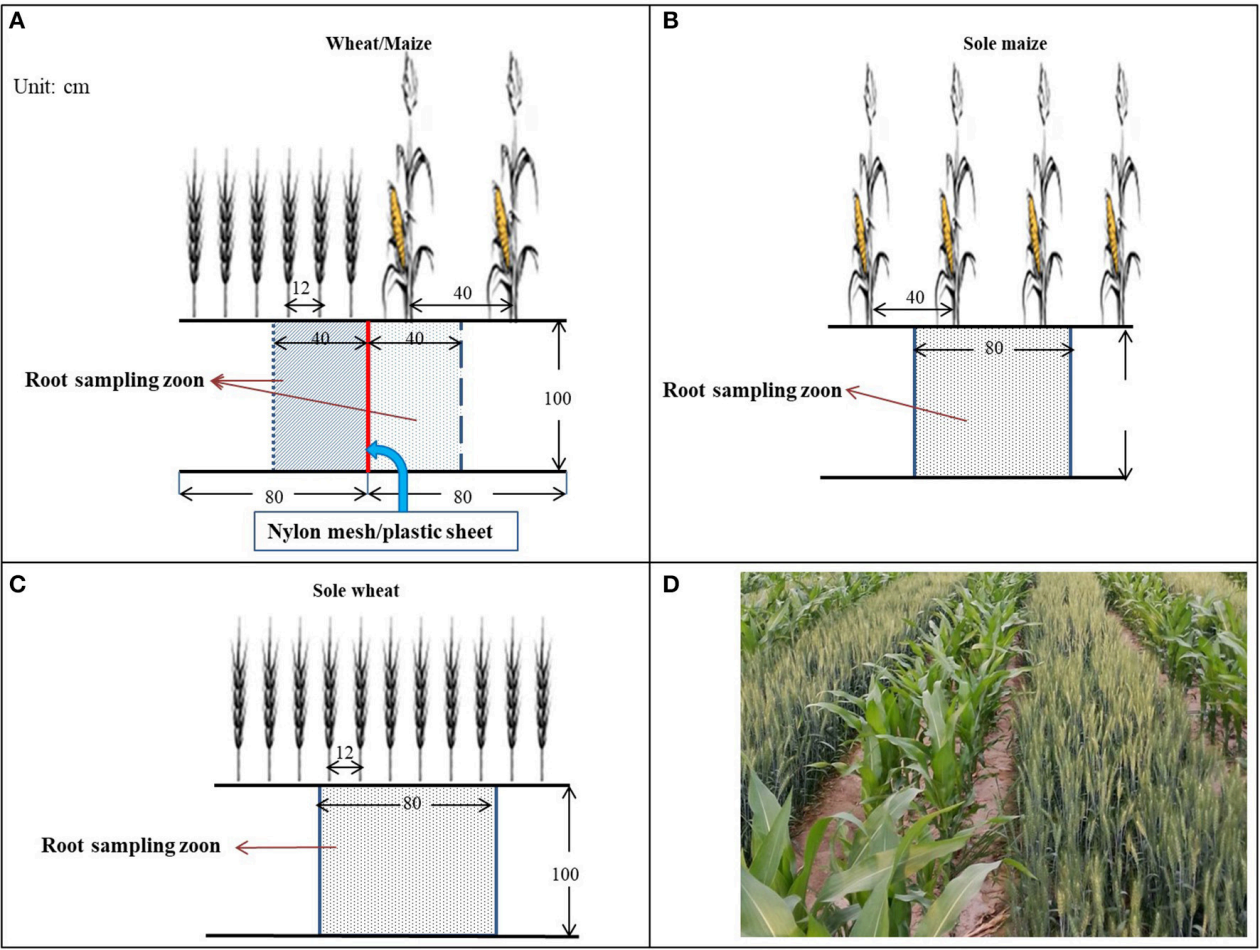
Layout of intercrops and the partition of roots in the wheat/maize intercropping system, with **(A)** wheat/maize intercropping with an 80-cm strip of wheat (six rows) alternated with an 80-cm strip of maize (two rows) in comparison with **(B)** single maize and **(C)** single wheat cropping. In the wheat/maize intercropping **(A)**, three root barrier treatments were included: (i) without a physical barrier (IC), (ii) with a nylon mesh barrier (IC-PRI), and (iii) with a solid plastic sheet (IC-NRI) inserted between wheat and maize strips. Nylon mesh and plastic sheet were placed vertically to the depth of 100 cm. The shadow section is the root sampling zone, and **(D)** shows the co-growth period of the wheat/maize strip intercropping in northwestern China.

A scheme of the field layout is shown in Figure 2. Each plot was 8 m long × 4.8 m wide in size. In the intercropping treatments, wheat and maize were planted in alternate strips with six rows of wheat (12-cm row spacing) alternated with two rows of maize (40-cm row spacing). Each plot contained three sets of wheat and maize strips. Prior to sowing, a trench (1 m deep, 0.1 m wide, and 8 m long) was made manually between the two crop strips, and the root barrier materials (nylon mesh with 300 micropores cm^−2^, or a flexible plastic sheet in 0.12 mm thickness) were vertically inserted into each trench and then filled with the excavated soil.

The sowing date and growth periods of spring wheat (cultivar Yong-Liang no. 4) and maize (cultivar Xian-Yu 335) are summarized in Table 1. All the strips of maize were covered with white plastic film at sowing, a practice widely adopted in the arid and semiarid northwest China for boosting maize productivity (Gan et al., 2013). Single wheat and single maize cropping received urea N-fertilizer at the rate of 225 and 450 kg N ha^−1^, respectively; and phosphorus oxide (P_2_O_5_) at 150 and 225 kg P ha^−1^, respectively. The fertilizer rates for the intercrops were the same as those for the single crops per area basis. Both N and P were applied at seeding for wheat, while a split application was used for maize crops, with 135 kg N ha^−1^ and 67.5 kg P ha^−1^ applied at seeding, 270 kg N ha^−1^ and 135 kg P ha^−1^ at stem elongation, and 45 kg N ha^−1^ and 22.5 kg P ha^−1^ at grain filling. The fertilization at seeding was implemented by broadcasting them on the soil surface and then incorporated to the soil using a shallow rotary tillage. Top-dressing fertilizers during the growth stages were implemented by making a hole of 10–15 cm deep and 5–6 cm away from the plant, a pre-determined amount of fertilizers was placed in the hole and the hole was filled with the soil. Crops were irrigated multiple times during the growing season (Table 2).

**Table 1:** Phenological stages of maize and wheat plants, at Wuwei Experimental Station, China, 2014, 2015, and 2016.

**Year**	**Crop**	**Sowing**	**Stem elongation/branching**	**Flowering/tasseling**	**Filling**	**Harvesting**
2014	Maize	Apr 22^a^	Jun 14	Jul 15	Aug 12	Oct 4
	Wheat	Mar 22	May 3	Jun 18	Jun 26	Jul 26
2015	Maize	Apr 25	Jun 16	Jul 16	Aug 12	Sep 28
	Wheat	Mar 28	May 5	Jun 18	Jun 26	Jul 27
2016	Maize	Apr 23	Jun 16	Jul 19	Aug 14	Sep 20
	Wheat	Mar 29	May 7	Jun 16	Jun 20	Jul 22

a *The dates of the phonological stage were recorded when 75% of the plants in a plot had developed to the particular growth stage*.

**Table 2:** The dates and the amounts of irrigation water applied to the wheat and maize, at Wuwei Experimental Station, China, 2014, 2015, and 2016.

**Year**	**Irrigation date**					
2014	May 5	May 30	Jun 20	Jul 30	Aug 19	Sep 5
2015	May 9	May 28	Jun 21	Jul 30	Aug 16	Sep 3
2016	May 8	May 27	Jun 16	Aug 1	Aug 18	Sep 2
**Cropping pattern**^a^	**Irrigation amount in each year (m**^3^ **ha**^−1^**)**					
W	750	900	750	0	0	0
M	750	900	750	900	750	750
IC	750	900	750	900	750	750

a *W, single-cropped wheat; M, single-cropped maize; IC, intercropped wheat/maize. The total amount of irrigation water is the sum of all irrigations during the entire growing season, i.e., the total amount was 2,400 m^3^ ha^−1^ for single-cropped wheat, 4,800 m^3^ ha^−1^ for single-cropped maize, and 4,800 m^3^ ha^−1^ for the wheat-maize intercropping*.

### Root sample collection

In arid and semiarid areas, the root growth of spring wheat often reaches the peak at flowering and then the rate of the growth may level off or decline. During the vigorous growth period, intercropped wheat plants have a competitive advantage for resources over the intercropped maize due to the earlier sowing of wheat than maize. However, after wheat harvest, a rapid recovery of the growth of the intercropped maize is highly expected due to more space and resources are available. Positive outcome of the growth recovery of intercropped maize can be realized during the grain filling period. Studies have shown large differences in maize root growth during grain filling compared with during the co-growth period (Li et al., 2001b). Therefore, in the present study, we sampled plant roots at wheat flowering and maize stem elongation stage (BCCH scale = 30) on 14 June in 2014, 17 June in 2015, and 12 June in 2016 for the first sampling. The second sampling was at the maize grain-filling stage (BCCH = 71) on 8 August in 2014, 11 August in 2015, and 13 August in 2016.

Plant roots were sampled using a modified monolith method (Smit et al., 2000) in the following three steps. First - preparing sampling trenches. A trench (100 cm long, 60 cm wide, and 120 cm deep) was made manually in each plot, with the direction of the trench length perpendicular to the crop row. Each trench covered an area of three rows of wheat and one row of maize in intercropping, or six rows of wheat or two rows of maize in the monoculture planting patterns. The readily-made trench provided sufficient space that allowed the sampler to work directly with the root-soil matrix with sampling tools. Second–marking the monoliths. The surface of the root-soil profile was smoothed by hand, and the size of each monolith to be sampled was marked on the profile wall using color makers. Each root-soil monolith was 40 cm long × 20 cm wide × 20 deep for each of the two intercrops, whereas it was 80 cm long × 20 cm wide × 20 deep for the monoculture planting patterns. The marking system clearly depicted the five monoliths to be sampled in each root-soil profile. For the intercropping, the five monoliths were marked in the intercropped wheat and the other five in the intercropped maize. Each monolith had a depth of 20 cm, and thus, the five monoliths had the total depth of 100 cm (Figure 2). Third–taking the monoliths. From the top layer of the profile, each monolith was cut following the marked lines using a sharp knife, a metal sheet sharpened on one side was inserted horizontally 40 cm onward into the profile, and the entire monolith was taken.

### Root weight density, root length density, and root surface density

Each root-soil monolith was placed in a 0.2-mm mesh bag, soaked in water for 1 h, gently stirred, and hand-scrubbed to clean the soil. The remaining debris was removed from the roots by hand. In wheat and maize intercropping with no root barrier, some monoliths contained the roots of both wheat and maize plants, and therefore it was necessary to distinguish between wheat and maize roots based on their visual appearance. Maize roots mostly had a larger diameter than wheat roots and some fine maize roots were yellowish, fragile, and with some visible nodes. The separated root fractions were placed into valve bags and brought to the laboratory. Root length and root surface area were immediately measured using an EPSON scanner in conjunction with Win-RHIZO^TM^ image analysis software (Régent Instruments Inc., Québec, Canada). The roots were then oven baked for 30 min at 105°C to deactivate enzymes, and thereafter were dried at 80°C until a constant weight and weighed for biomass with an accuracy of 0.001 g. Finally, the root weight density (RWD: grams of dry root weight per cubic centimeter of the monolith), root length density (RLD: centimeter of root length per cubic centimeter), and root surface area density (RSAD: square centimeter of root surface area per cubic centimeter) were calculated from the volume of the soil monolith using the following formulae:

(1) RWD=RW/SV

(2) RLD=RL/SV

(3) RSAD=RSA/SV

where *RW* is root dry weight, *RL* is root length, *RSA* is root surface area, and *SV* is soil volume. Due to the sample size, the soil volume of the intercropped wheat and intercropped maize each was 16,000 cm^3^, whereas the volume of single-cropped wheat and maize each was 32,000 cm^3^.

For each of the three replicates, the total RWD of the 0–100-cm soil layer was the sum of the weight of the roots from each of the depths and divided by the combined volume of the individual compartments. The average was based on the three replicates. The calculation of the averages of RLD and RSAD was the same as that used for the calculation of RWD.

### Methods for calculating indices

#### Recovery effect

Interspecific competition may occur when two crops are grown together or in neighboring strips (Vandermeer, 1989). Such competition generally decreases the growth of at least one of the intercropped species (Yin et al., 2017). In the case of wheat-maize intercropping, the wheat often inhibits the growth of maize during the co-growth period. After harvest of the early-maturing wheat, the remaining intercropped maize accelerates the growth to form a compensatory growth period. The recovery process in maize plant growth could offset the impaired early growth occurring during the co-growth period (Zhang and Li, 2003; Li L. et al., 2011). The roots of the intercropped maize that occupy a large belowground space may play an important role in the recovery growth (Li L. et al., 2011). In the present study, we defined the recovery effect of the intercropped maize (RE) as the differences of the root growth rate between the intercropped maize and the corresponding monoculture maize as follow:

(4) $$RE=RGR_{I}/RGR_{M}$$

where *RGR*_*I*_ is the root growth rate of the intercrops, and *RGR*_*M*_ is the root growth rate of the monoculture maize.

The root growth rate was defined as:

(5) $$RGR=(R_{2}-R_{1})/(t_{2}-t_{1})$$

where *R*_2_ and *R*_1_ is the root traits measured at the two consecutive time *t*_2_ and *t*_1_, respectively. The *t*_2_ sampling was at the maize filling stage and the *t*_1_ sampling was at the wheat flowering stage or at the maize stem elongation stage. The calendar dates of samplings differed slightly each year and were detailed in Table 1. The RGR was determined for each of the three key root traits: RWD, RLD, and root surface area density, in the 0–100 cm profile. An RE value >1.0 indicates that intercropped maize had a recovery effect after the harvest of the co-cropped wheat (Yin et al., 2017).

#### Grain yield, biomass, and harvest index

For both single-cropping and the intercropping systems, the maize and wheat in each plot was hand-harvested at full maturity, and the samples of grains and straw were air dried, cleaned, and weighed to determine grain and biomass yields (BYs), respectively. The percentage of yield increase of intercropping compared with sole cropping is equal to the total yield of intercropping system divided by the yield of the corresponding sole crops. All yields were expressed on a per unit area basis. The term “overyielding” was used to quantify the magnitude of the yield increase for each of the two intercrops over the corresponding sole crops.

Overyielding for grain was calculated by following the formula developed by Li Q. Z. et al. (2011):

(6) $$Overyielding(\%)=\frac{Y_{intercrop}\;-\;Y_{solecrop}\;\times \;Pr}{Y_{solecrop}\;\times \;Pr}\times 100$$

where *Y*_*Intercrop*_ is the yield of the intercrop (wheat or maize), *Y*_*solecrop*_ is the yield of sole crop (wheat or maize), *Pr* is the land area occupied by each intercrop in proportion to total area. In this study, the *Pr*_*wheat*_ = *Pr*_*maize*_ = 50%. Overyielding for the intercropping was assessed by an increase or decrease in the intercropped crops over the corresponding single crop. A positive overyielding indicates a yield advantage for the intercropping over the corresponding single crop.

The harvest index (HI) was determined by dividing the grain yield (GY) by the aboveground biomass yield (BY) per unit area.

(7) HI = GY/BY

#### Three root barrier treatments and possible interspecific interactions

The three aforementioned root barrier treatments were implemented in each year. The no-barrier treatment allowed intercrop roots to penetrate into neighboring strips and freely exchange water and nutrients, whereas the nylon mesh barrier physically blocked root penetration but allowed potential movement of water and nutrients between the plant rows. In contrast, in the plastic sheet barrier treatment, the solid plastic sheet separated the two intercrops physically, preventing any belowground interspecies interaction. Therefore, a comparison between the no barrier (complete belowground interaction, IC) and plastic sheet barrier (no root interaction, only aboveground interaction, IC-NRI) treatments enabled us to determine the contribution of belowground interspecific interaction. Similarly, a comparison between the no barrier (IC) and nylon mesh barrier (partial belowground interaction, IC-PRI) treatments enabled us to determine the compensational effect due to root overlap between the two intercrops; a comparison between nylon-mesh barrier (IC-PRI) and plastic sheet barrier (IC-NRI) treatments enabled us to determine the compensational effect due to water and nutrient exchange (CWN).

To quantify the belowground interspecies interaction, we determined the contribution rate of belowground interaction (CRB) as follows:

(8) $$CRB(\%)\;=\;\frac{Y_{IC}\;-\;Y_{IC-NRI}}{Y_{IC-NRI}}\;\times \;100$$

where *Y*_*IC*_ and *Y*_*IC*−*NRI*_ are the GY or recovery effect of intercropping without a barrier and intercropping with a plastic sheet barrier, respectively.

The compensational effect due to root overlapping (CRO) was calculated as follows:

(9) $$CRO(\%)\;=\;\frac{Y_{IC}\;-\;Y_{IC-PRI}}{Y_{IC-PRI}}\;\times \;100$$

where *Y*_*IC*_ and *Y*_*IC*−*PRI*_ are the GY or recovery effect of intercropping without a barrier and intercropping with a nylon mesh barrier, respectively.

The CWN was calculated as follows:

(10) $$CWN(\%)\;=\;\frac{Y_{IC-PRI}\;-\;Y_{IC-NRI}}{Y_{IC-NRI}}\;\times \;100$$

where *Y*_*IC*−*PRI*_ and *Y*_*IC*−*NRI*_ are the same as defined above.

#### Statistical analysis

The data were analyzed using the Statistical Package for Sciences statistical analysis software (SPSS software, 19.0; SPSS Inst. Ltd., USA). Two–way analysis of variance (ANOVA) followed by the Fisher Protected Least Significance Difference (LSD) test was performed to determine the main effects (belowground interaction pattern and maize density) and their interactions on the variables RWD, RLD, RSAD, RE, and GY. When belowground interaction pattern × plant density interaction effect was not significant, the means of belowground interaction patterns and the means of densities were presented; however, when belowground interaction pattern × density interaction was significant, the belowground interaction effect was determined for each of the two maize density levels, and density effect was determined at each of the three belowground interaction treatments. The significances between treatments were determined using the LSD multiple-range test at the 0.05 probability level. Due to significant treatment by year interactions for most of the variables, the treatment effects are presented separately for each year. However, the root growth rate and recovery effect are presented as the 3-year averages because they did not differ significantly between the study years.

## Results

### Wheat root development in relation to belowground interaction during the co-growth period

**Figure 3:**
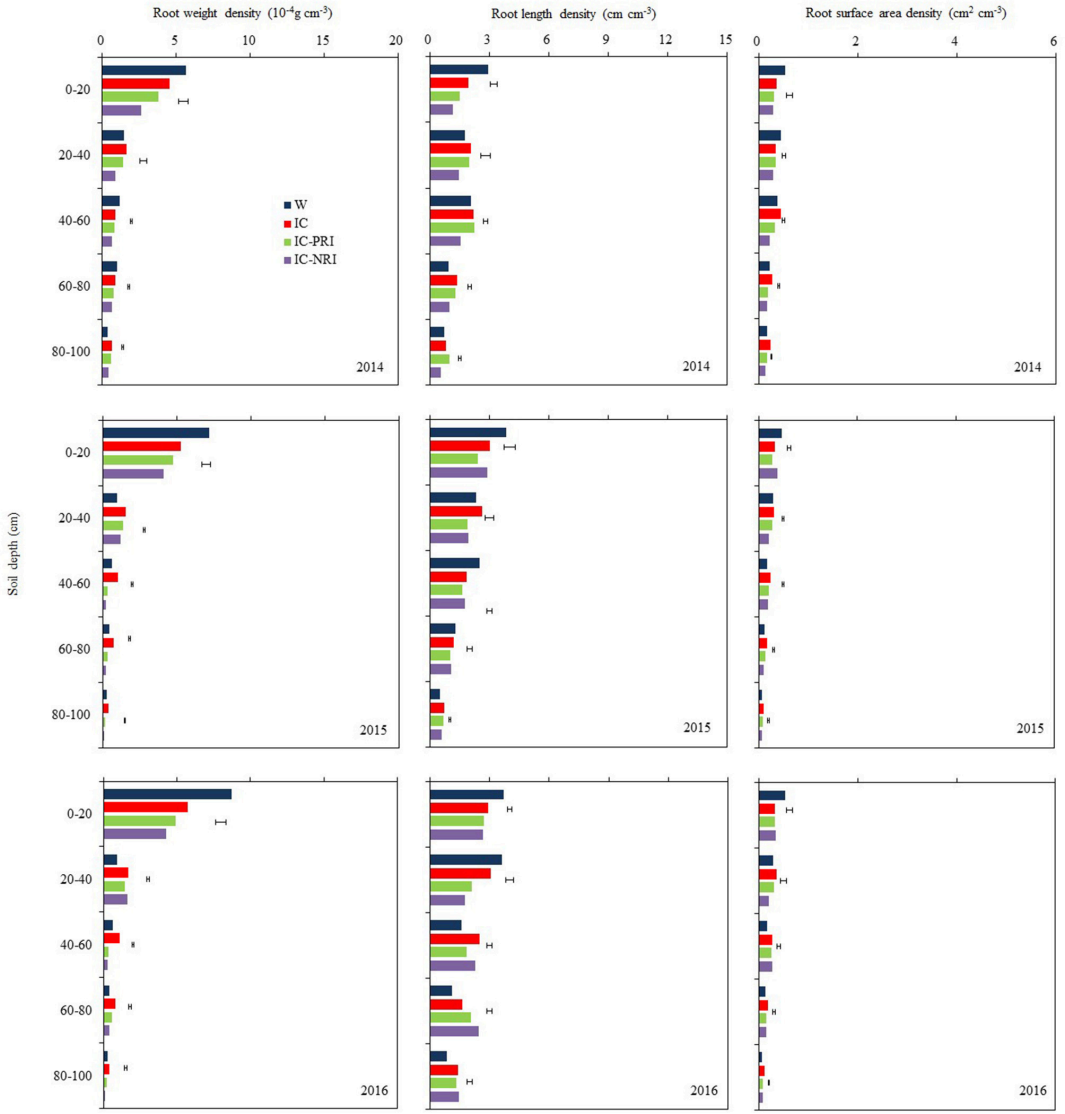
The root weight density (RWD), root length density (RLD), and root surface area density (RSAD) of wheat at the wheat flowering stage, at Wuwei Experimental Station, in 2014, 2015, and 2016. W, single-cropped wheat; IC, wheat and maize intercropping with no root restrictions (i.e., no root barrier); IC-PRI, wheat and maize intercropping with partial root interaction (i.e., nylon mesh root barrier); IC-NRI, wheat and maize intercropping with no root interaction (i.e., plastic sheet root barrier); The line bars are least significant differences (LSDs), at the *P* < 0.05 level, among the different treatments within each soil layer.

Wheat roots were sampled in mid-June each year, which coincided with a time when the wheat plants were at the mid-flowering stage (Figure 3). During this period, most of wheat roots were distributed in the 0–20 and 20–40-cm soil layers. For single-cropped wheat, 72% of roots were distributed in the 0–20-cm soil layer and 11% in the 20–40-cm soil layer, whereas for intercropped wheat, 57% of roots were distributed in the 0–20-cm soil layer and 20% were in the 20–40-cm soil layer. Wheat in the treatments IC-PRI (partial belowground interaction) and IC-NRI (without belowground interaction) had lower total RWD, RLD, and root surface area (RSAD) compared with single-cropped wheat in the 0–100-cm soil layer. However, single-cropped wheat and the intercropped wheat in the complete belowground interaction (IC, without root barrier) had similar total (0–100 cm) RLD, and RSAD. The IC treatment had a lower RWD in the 0–20-cm soil layer than the single-cropped wheat, but the former had a significantly higher RWD than the single-cropped wheat in the 20–100 cm soil layer.

The treatment × year interaction was significant for RWD most likely due to the complex nature of many soil layers in which the measurements were taken. However, the trend of the treatment effects was similar across the 3 study years (Figure 3). Also, maize density had no effect on the RWD of the intercropped wheat. Thus, the averages of the treatment effects over the two densities across the three years were discussed. The IC treatment with complete belowground interaction increased the RWD of intercropped wheat significantly. In the 0–20, 20–40, 40–60, 60–80, and 80–100-cm soil layers, the IC treatment increased RWD by 45, 36, 231, 131, and 222%, respectively, compared with the IC-NRI treatment; and the value of the increase was 16, 16, 142, 63, and 78% compared with the IC-PRI treatments.

Maize density had no effect on the RLD of the intercropped wheat (Figure 3). The IC treatment increased the RLD of intercropped wheat compared with the IC-PRI and IC-NRI treatments. In the 0–20, 20–40, and 40–60-cm soil layer, the IC treatment increased wheat RLD by 69, 43, and 44% in 2014 and by 9, 72, and 11% in 2016, compared with the IC-NRI treatment. However, no differences were found between those intercropping treatments in 2015. In the 60–80 and 80–100-cm soil layers, the IC treatment increased wheat RLD by 37 and 50% in 2014 and by 11 and 20% in 2015, compared with IC-NRI treatment, with no differences found in 2016. Similarly, in the 0–20-cm soil layer, wheat RLD was improved by 29% in 2014 and by 25% in 2015 under the IC treatment, compared with the IC-PRI treatment, but no difference was found in 2016. In the 20–40 and 40–60-cm soil layers, wheat RLD was improved by 38 and 13% in 2015 and by 44 and 37% in 2016 under IC treatment, compared with the IC-PRI treatment, with no differences found in 2015. In the 60–100 cm soil layer, there was no significant difference between IC and IC-PRI treatment.

Maize density had no effect on the RSAD of the intercropped wheat (Figure 3). The RSAD of intercropped wheat was reduced by 20% in 2015 and 10% in 2016 under IC treatment, compared with that in the IC-NRI treatment, with no difference found in 2014. However, on three year average, in the 20–40, 40–60, 60–80, and 80–100-cm soil layers, wheat RSAD in the IC treatment was, respectively, 51, 42, 54, and 65% greater than wheat in the IC-NRI; and was, respectively, 11, 17, 37, and 32% greater than wheat in the IC-PRI treatment.

### Maize root development in relation to belowground interactions during the co-growth period

**Figure 4:**
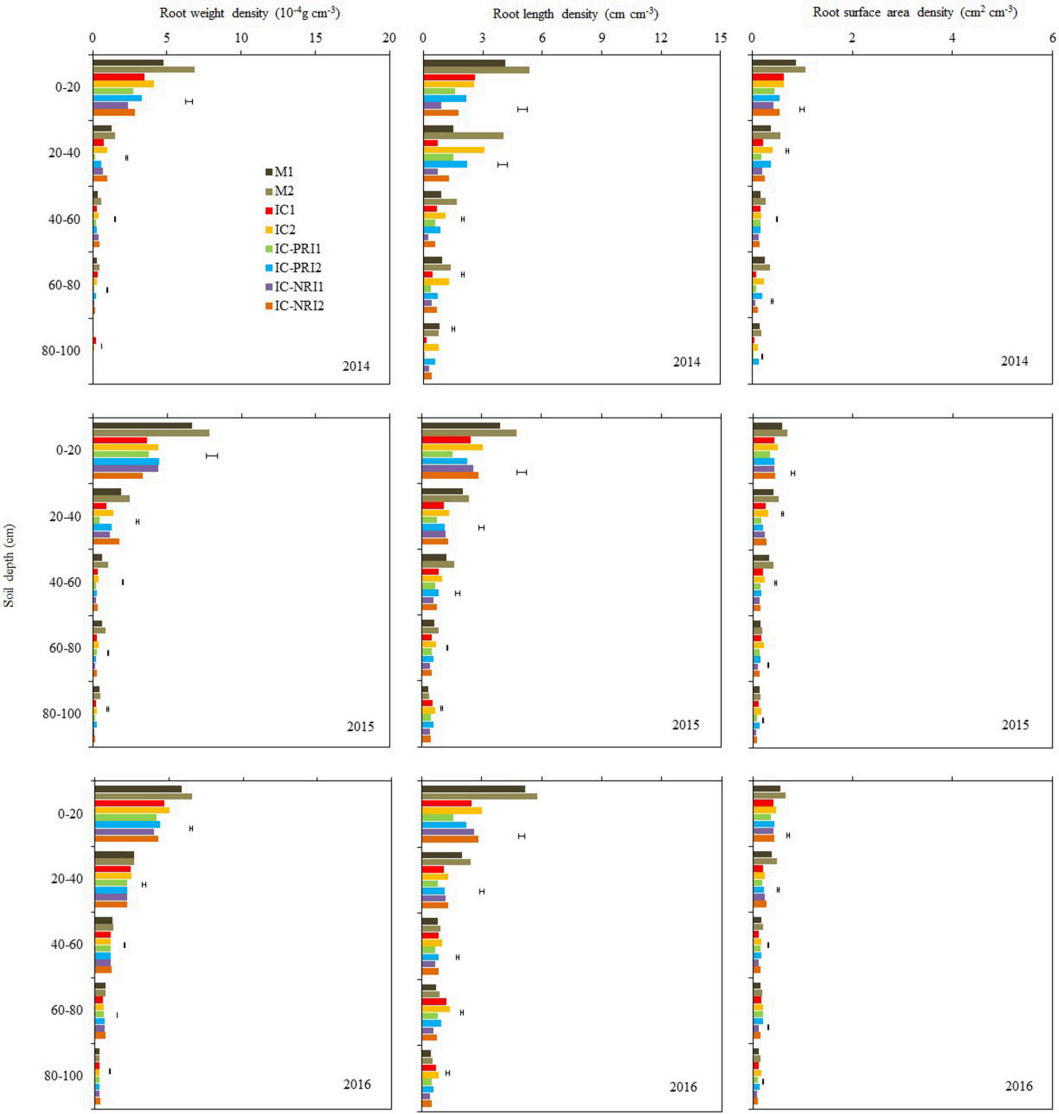
The root weight density (RWD), root length density (RLD), and root surface area density (RSAD) of maize at the maize stem elongation stage, at Wuwei Experimental Station, China, 2014, 2015, and 2016. M single-cropped maize; IC, wheat and maize intercropping with no root restrictions (i.e., no root barrier); IC-PRI, wheat and maize intercropping with partial root interaction (i.e., nylon mesh root barrier); IC-NRI, wheat and maize intercropping with no root interaction (i.e., plastic sheet root barrier); “1” and “2,” low and high density of maize plants, respectively. The line bars are least significant differences (LSDs), at the *P* < 0.05 level, among the different treatments within each soil layer.

Maize roots were sampled at the beginning of stem elongation (BBCH = 30), a time when the intercropped wheat was at the mid-flowering stage (Figure 4). At this stage, about 70% of the roots in single-cropped maize were distributed in the 0–20-cm soil layer and about 20% in the 20–40-cm soil layers, whereas about 60% of the roots in the intercropped maize were distributed in the 0–20 cm soil layer and about 20% in the 20–40-cm soil layers. Single-cropped maize had higher total RWD, RLD, and RSAD than the intercropped maize in most of the soil layers. Single-cropped maize had higher total RWD, RLD, and RSAD compared with the intercropped maize in the 0–100-cm soil layer. Averaged over the three years, single-cropped maize increased total RWD, RLD, and RSAD by 53, 81, and 70%, respectively, than the maize in the IC treatment; the values of the increases were 49, 96, and 109% compared with the maize in the IC-PRI treatment, and were 43, 69, and 82% compared with the maize in the IC-NRI treatments.

The three belowground interaction treatments had significant effects on the RWD of intercropped maize in most of the soil layers (Figure 4). In the 0–20-cm soil layer, the maize RWD in the IC treatment was 45% greater than that in the IC-NRI treatment in 2014 and 18% greater in 2016, but no difference was found in 2015. In the 60–80 and 80–100-cm soil layers, the IC treatment increased maize RWD by 117 and 183%, respectively for the two soil depths in 2014 and 38 and 145% in 2015, compared with those in the IC-NRI treatment. However, in 2016, maize RWD did not differ between the treatments. In the 0–20 and 20–40 cm soil layers, the IC treatment increased maize RWD by 25% and 126%, respectively for the two soil depths in 2014 and 13 and 13% in 2016, but no differences were found in 2015, as compared with those in the IC-PRI treatment. Also, in the 40–60, 60–80, and 80–100-cm soil layers, the IC treatment increased the maize RWD by 35, 103, and 240%, respectively in 2014; 35, 26, and 36% in 2015, compared with the IC-PRI treatment, but no differences were found in 2016. Maize plant density had an interactive effect on maize RWD. With the maize density increase from the low to high, the RWD in the 20–40, 40–60, and 60–80-cm soil layers increased, respectively, by 52, 32, and 23% in 2014; and 77, 131, and 21% in 2015. In 2016, however, there was no significant difference between high and low maize densities in affecting RWD for either of the soil layers. Complete belowground interaction with the high maize density increased the RWD of intercropped in the 60–80-cm soil layer compared with the low maize density.

The response of maize RLD to the interspecies interaction treatments followed a similar trend as the effect on RWD but the treatment by year interaction on RLD was not significant, thus the average RLD across the three years are discussed here. In the 40–60, 60–80, and 80–100-cm soil layers, the IC treatment increased maize RLD by 22, 44, and 33%, respectively, as compared with the IC-PRI treatment (Figure 4); the maize RLD under the IC treatment was 60, 65, and 48% greater than those under the IC-NRI treatment, respectively, for the three soil depths. In the 0–20 and 20–40-cm soil layer, the maize RLD in the IC treatment did not differ from that in the IC-NPR treatment, but increased by 42% in the 0–20 soil layer and 28% in the 20–40 cm soil layer compared with the IC-PRI treatment. The maize plants under the high density increased RLD by 24, 56, 40, 52, and 95%, respectively, in the 0–20, 20–40, 40–60, 60–80, and 80–100-cm soil layers, compared to the low plant density. The treatment of complete interspecies interaction in combination with high maize plant density achieved the highest RLD in the 40–100-cm soil layer among all the treatments.

Complete belowground interactions in combination with high maize density significantly increased the RSAD in the most of the soil layers (Figure 4). On average, the maize in the IC treatment increased RSAD by 36, 59, and 197%, respectively, in the 40–60, 60–80, and 80–100-cm soil layer, compared with the IC-NRI treatment; the maize in the IC-PRI treatment increased RSAD by 13, 39, and 175% in the three respective depths, compared with the IC-NRI treatment. However, in the 0–20 and 20–40-cm soil layers, the values of maize RSAD did not differ among the IC, IC-PRI, and IC-NRI treatments. The maize at the high density increased the RSAD by 14, 33, 17, 59, and 108%, respectively, in the 0–20, 20–40, 40–60, 60–80, and 80–100-cm soil layers, compared with the low density. The treatment of complete belowground interaction in combination with high maize density achieved the highest RSAD values in the 40–100-cm soil layer among all treatments.

### Maize root development in relation to belowground interactions after wheat harvest

**Figure 5:**
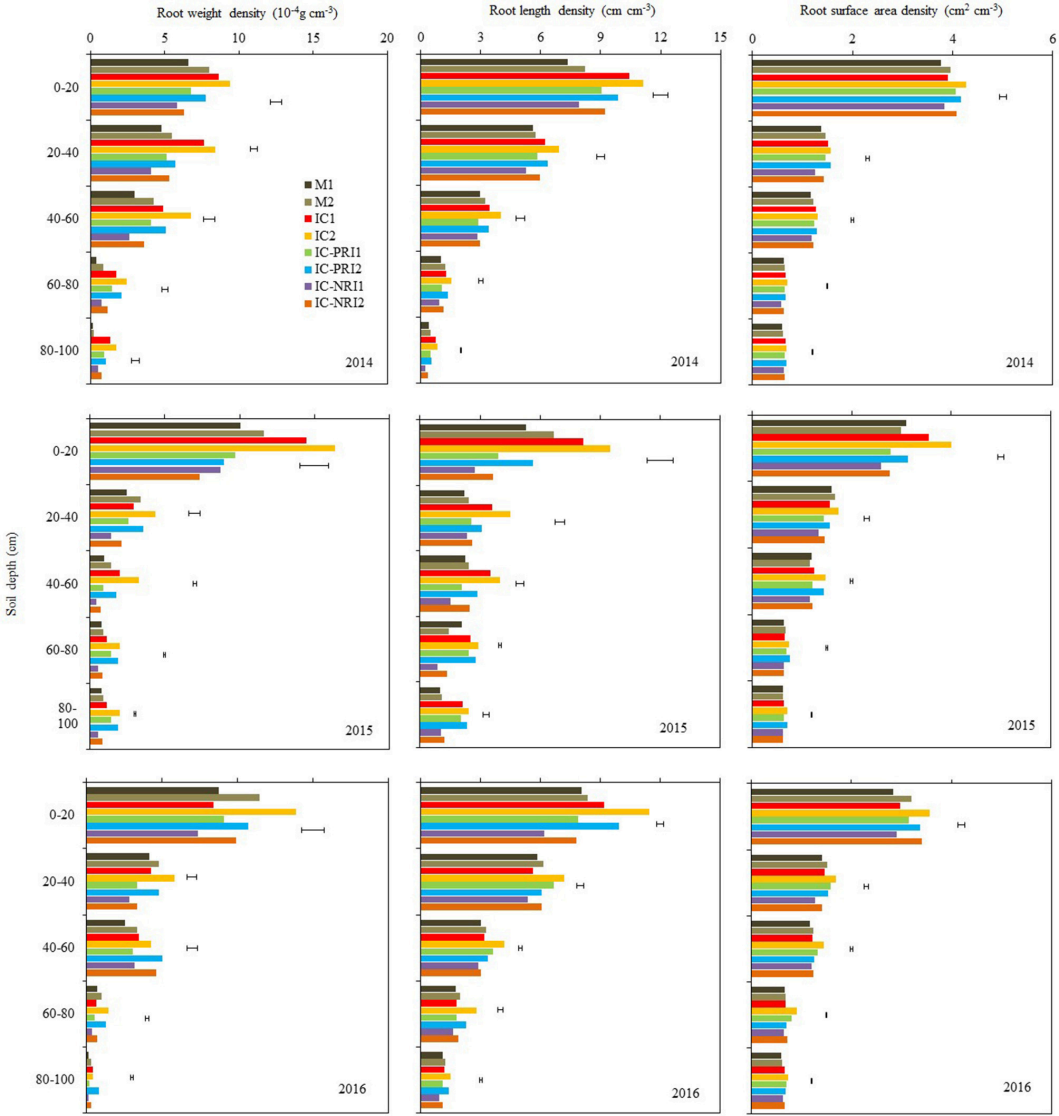
The root weight density (RWD), root length density (RLD), and root surface area density (RSAD) of maize at the maize grain-filling stage, at Wuwei Experimental Station, China, 2014, 2015, and 2016. M single-cropped maize; IC, wheat and maize intercropping with no root restrictions (i.e., no root barrier); IC-PRI, wheat and maize intercropping with partial root interaction (i.e., nylon mesh root barrier); IC-NRI, wheat and maize intercropping with no root interaction (i.e., plastic sheet root barrier); “1” and “2,” low and high density of maize plants, respectively. The line bars are least significant differences (LSDs), at the *P* < 0.05 level, among the different treatments within each soil layer.

Maize roots were sampled for the second time at the beginning of grain development (BBCH = 71), about 20 days after the wheat had been harvested. At this stage, the maize plants in the IC treatment had greater RWD, RLD, and RSAD compared with single-cropped maize (Figure 5). Averaged across the three study years, the maize in the IC treatment increased RWD, RLD, and RSAD by 40, 44, and 11%, respectively, compared with single-cropped maize, whereas there were no significant differences in these values between the single-cropped maize and the IC-PRI or IC-NRI treatments.

Complete belowground interactions and increasing maize density increased RWD in the 0–80-cm soil layer (Figure 5). On the three year average, the maize RWD in the IC treatment was 34, 30, 62, and 17% greater than those in the IC-PRI treatment, respectively, in the 0–20, 20–40, and 40–60, and 60–80-cm soil layers; and they were 50, 81, 221, 116, and 104% greater than those in the IC-NRI treatments, respectively, In the 80–100-cm soil layer, there was no significant difference between the IC and IC-PRI treatments, whereas the RWD in the IC treatment was increased by 104% compared with the IC-NRI treatment. The maize under the high density increased RWD in the 0–20 soil layers by 10% in 2014 and 51% in 2016, but no difference was found in 2015. In the 20–40, 40–60, 60–80, and 80–100-cm soil layers, the high density increased the maize RWD by 31, 50, 76, and 55%, respectively, on the average of three years. The complete belowground interaction, in combination with high maize density, led to the highest RWD in the 0–80-cm soil layer, among all the treatments.

There was a significant interaction of belowground interaction treatment and maize density in affecting RLD of the intercropped maize but the effect followed a similar trend in each year. Averaged across the three year, RLD in the IC1 treatment was 73, 26, 54, 80, and 123% greater than that in the IC-NRI1 treatment in the 0–20, 20–40, 40–60, 60–80, and 80–100-cm soil layers, respectively (Figure 5). Similarly, RLD in the IC2 treatment was 62, 35, 44, 66, and 89% greater than that in the IC-NRI1 treatment in the five respective soil layers. There was no significant difference in RLD between the IC-PRI and IC-NRI treatments in the 0–100-cm soil layers. In the 0–20, 20–40, 40–60, 60–80, and 80–100-cm soil layer, the maize in the IC treatment with the high density increased RLD by 16, 21, 20, 32, and 17%, respectively, compared with the low density; similarly, the high-density maize in the IC-PRI treatment increased RLD by 15, 7, 16, 23, and 18%; and by 19, 13, 24, 33, and 33% in the IC-NRI treatment, compared with the low density. The high density with the complete belowground interaction had the highest RLD in the 0–100-cm soil layer among all the treatments.

Complete belowground interactions and increasing maize density increased RSAD (Figure 5). There was no significant difference in RSAD between the IC and IC-PRI treatments, both being higher than that in the IC-NRI treatment. On three-year averages, in the 20–40, 40–60, 60–80, and 80–100 cm soil layer, the RSAD in the IC treatment was, respectively, 16, 10, 13, and 8% higher compared with the IC-NRI treatment. Similarly, the RSAD in the IC-PRI treatment was 12 and 11% higher, respectively, in the 20–40 and 60–80-cm soil layers, compared with the IC-NRI treatment. With the increase in maize density, the maize RSAD increased by 11% only in the 0–20 soil layers, across the three years. Thus, complete belowground interaction with the high maize density improved the RSAD of intercropped maize in the 0–20-cm soil layer.

### Root growth and recovery of maize after wheat harvest

From the stem elongation to the grain-filling stage of maize, the roots of intercropped maize grew rapidly. Comparisons of the root growth rates (RGR) of the intercropped maize with those of single-cropped maize revealed that, after wheat harvest, intercropped maize had a significantly higher RGR than single-cropped maize (Table 3). Averaged across the three study years, the intercropped maize increased RGR by 138, 85, and 11% for RWD; by 110, 105, and 58% for RLD; and by 22, 22, and 12% for RSAD, in the IC, IC-PRI, and IC-NRI treatments, respectively, compared with the single-cropped maize. In general, the RGR for RWD, RLD, and RSAD increased by the largest percentage under complete belowground interaction. With the increase in maize plant density, the RGR for RWD increased proportionally. The RGR for RWD in the M, IC, IC-PRI, and IC-NRI treatments increased by 31, 29, 33, and 43%, respectively, with the high maize density. The RGR for RLD and RSAD tended to increase less. Thus, intercropping with complete belowground interaction at a higher planting density promoted RGR.

**Table 3:** Root growth rate (RGR) of maize plants from stem elongation (BBCH 31) to the grain filling (BBCH 71) under different treatments, with the root characteristics reflected by the root weight density (RWD), root length density (RLD), and root surface area density.

**Treatment^a^**	**Root growth rate for the variable^b^**		
	**RWD**	**RLD**	**RSAD**
	**(10^−4^g cm^−3^ d^−1^)**	**(cm cm^−3^ d^−1^)**	**(cm^2^ cm^−3^ d^−1^)**
M1	0.31	0.41	0.28
M2	0.40	0.34	0.27
IC1	0.74	0.78	0.32
IC2	0.95	0.79	0.34
IC-PRI1	0.56	0.73	0.33
IC-PRI2	0.75	0.79	0.34
IC-NRI1	0.33	0.56	0.30
IC-NR2	0.46	0.62	0.31
*p*-value^c^	<0.01	<0.01	<0.01
LSD (0.05)	0.02	0.04	0.004

a *W, single-cropped wheat; M single-cropped maize; IC, intercropped wheat and maize with no root restrictions (i.e., no root barrier); IC-PRI, intercropped wheat and maize with partial root interaction (i.e., nylon mesh barrier); IC-NRI, intercropped wheat and maize with no root interaction (i.e., plastic sheet barrier); “1” and “2,” low and high density of maize plants, respectively*.

b *The root growth rate reflected by the root weight density, root length density, and root area surface density for the total soil profile (0–100 cm); the values are averages of 2014, 2015, and 2016 because of no significant year × treatment interactions*.

c *The p-value and the LSD (0.05) were for all the treatments in a column*.

As defined in Equation (4) above, the recovery effect was expressed as the RGR value of intercropped maize divided by the RGR in the single-cropped maize. In the presented study, the 3-year average recovery effect for intercropped maize was <1, indicating that intercropped maize had a recovery effect following the harvest of the co-cropped wheat. The recovery effect in the IC treatment resulted in a 143 and 35% increase in RWD compared with the IC-NRI and IC-PRI treatments, respectively (Table 4). Further, the recovery effect of RWD in the IC-PRI treatment was increased by 80% compared with the IC-NRI treatment. The recovery effect of RLD and RSAD showed trends similar to that of RWD. The recovery effect of RLD was affected by maize density. The recovery effect of RLD in the IC and IC-PRI treatments was increased by 30 and 37%, respectively, with the increased maize density. In general, the complete belowground interaction with the high maize density promoted the root recovery. On the average of the three study years, the contribution rate due to belowground interspecies interaction was 143%, the compensational effect due to root overlap was 35%, and the CWN was 80%, for the recovery effect of RWD.

**Table 4:** Recovery effect (RE) of maize plants after wheat harvest under different treatments, with the root characteristics reflected by the root weight density (RWD), root length density (RLD), and root surface area density (RSAD).

**Treatment^a^**	**Recovery effect for the variable^b^**		
	**RWD^a^**	**RLD**	**RSAD**
IC1	2.51	2.14	1.17
IC2	2.59	2.78	1.27
IC-PRI1	1.88	1.83	1.18
IC-PRI2	1.89	2.50	1.28
IC-NRI1	1.03	1.26	1.07
IC-NRI2	1.07	1.46	1.17
*p*-value^c^	<0.01	<0.01	<0.01
LSD (0.05)^b^	0.23	0.29	0.04

a *W, single-cropped wheat; M single-cropped maize; IC, intercropped wheat and maize with no root restrictions (i.e., no root barrier); IC-PRI, intercropped wheat and maize with partial root interaction (i.e., nylon mesh barrier); IC-NRI, intercropped wheat and maize with no root interaction (i.e., plastic sheet barrier); “1” and “2,” low and high density of maize plants, respectively*.

b *The recovery effect of root weight density, root length density, and root area surface density for all soil profile (0–100 cm); the values are averages of 2014, 2015, and 2016 because of no significant year x treatment interactions*.

c *The p-value and the LSD (0.05) were for all the treatments in a column*.

### Grain yield, biomass, and harvest index

Consistent grain yields were obtained over the three study years, with the intercropping systems having yield advantages over the corresponding single cropping (Table 5). On three years average, the total GY of intercropping was 137% greater compared with the corresponding single-cropped wheat, and was 14% greater compared with the single-cropped maize. The intercropped wheat produced 53% higher GY than the single-cropped wheat, and the intercropped maize produced 55% higher GY that the single-cropped maize. The trend in biomass of each treatment was similar to that of grain yield.

**Table 5:** Grain yield, biomass, and harvest index of wheat and maize in monoculture and wheat-maize intercropping under different root interaction treatments, at Wuwei Experimental Station, in 2014, 2015, and 2016.

	**Grain yield**			**Biomass**			**Harvest index**	
**Treatment**	**Wheat**	**Maize**	**Total**	**Wheat**	**Maize**	**Total**	**Wheat**	**Maize**
**2014**								
W	6,275	–	6,275	14,934	–	14,934	0.420	–
M1	–	11,501	11,501	–	23,765	23,765	–	0.484
M2	–	12,081	12,081	–	25,797	25,797	–	0.468
IC1	4,581	8,215	12,097	9,759	19,793	29,552	0.469	0.415
IC2	4,251	10,126	14,578	9,437	20,835	30,272	0.472	0.486
IC-PRI1	3,981	8,004	12,086	9,020	18,973	27,993	0.452	0.422
IC-PRI2	3,824	9,728	13,652	8,634	20,023	28,657	0.455	0.486
IC-NRI1	3,407	7,992	11,399	7,836	17,966	25,802	0.437	0.472
IC-NRI2	3,319	8,943	12,263	7,601	18,953	26,555	0.435	0.445
*P*-value^a^	<0.01	<0.01	<0.01	<0.01	<0.01	<0.01	<0.01	<0.01
LSD (0.05)	353	489	381	532	922	692	0.012	0.014
**2015**								
W	6,070	–	6,070	15,650	–	15,650	0.390	–
M1	–	13,795	13,795	–	30,112	30,112	–	0.467
M2	–	12,819	12,819	–	31,366	31,366	–	0.447
IC1	4,434	9,149	13,584	10,843	19,211	30,055	0.410	0.447
IC2	4,514	10,807	15,322	10,922	23,012	33,935	0.413	0.463
IC-PRI1	4,002	8,758	12,761	10,422	18,771	29,193	0.386	0.476
IC-PRI2	3,917	9,852	13,769	10,308	22,054	32,362	0.380	0.469
IC-NRI1	3,451	8,149	11,600	9,632	17,614	27,246	0.360	0.409
IC-NRI2	3,609	8,435	12,045	9,696	18,852	28,548	0.373	0.458
*P*-value^a^	<0.01	<0.01	<0.01	<0.01	<0.01	<0.01	<0.01	<0.01
LSD (0.05)	258	463	449	477	1,329	1,274	0.039	0.022
**2016**								
W	6,313	–	6,313	16,748	–	16,748	0.372	–
M1	–	12,407	12,408	–	40,613	40,613	–	0.306
M2	–	15,307	15,307	–	43,477	43,477	–	0.286
IC1	5,327	10,421	15,781	10,900	25,127	36,028	0.461	0.395
IC2	5,400	11,816	17,203	10,613	34,930	45,543	0.459	0.326
IC-PRI1	5,026	9,934	14,926	11,059	22,344	33,403	0.482	0.466
IC-PRI2	4,876	11,381	16,271	11,167	20,930	32,097	0.484	0.565
IC-NRI1	4,718	8,037	12,722	9,865	15,625	25,491	0.478	0.514
IC-NRI2	4,475	9,107	13,626	9,581	22,836	32,417	0.467	0.399
*p*-value^a^	<0.01	<0.01	<0.01	<0.01	<0.01	<0.01	<0.01	<0.01
LSD (0.05)	229	715	670	630	6,735	6,286	0.028	0.063

*W, single-cropped wheat; M single-cropped maize; IC, intercropped wheat and maize with no root restrictions (i.e., no root barrier); IC-PRI, intercropped wheat and maize with partial root interaction (i.e., nylon mesh barrier); IC-NRI, intercropped wheat and maize with no root interaction (i.e., plastic sheet barrier); “1” and “2,” low and high density of maize plants, respectively*.

a *The p-value and the LSD (0.05) are for all treatments in each year*.

*The unit of yield for the single-cropped wheat and single-cropped maize is kg ha^−1^, whereas for the intercropping systems, the unit of the yield for the intercropped wheat and intercropped maize is kg 0.5 ha^−1^ as each intercrop occupied ½ of the land area*.

Comparisons of the different belowground interaction patterns showed a significant yield increase with the complete belowground interaction in combination with the high maize density (Table 5). Averaged over the three study years, the complete belowground interaction contributed 20% GY increase, and the high density contributed 7% more GY than maize at the low density. Similarly, the CRO led to a 6% yield increase, and the effect was 4% greater with the high than the low maize density. The CWN led to a 13% yield increase, and the effect was 4% greater with the high than the low maize density.

Intercropped wheat had a greater HI with the higher maize density. The HI of wheat grown in the intercropping system was 12% greater in 2014 and 23% greater in 2016 compared with single-cropped wheat (Table 5). In contrast, the HI of intercropped maize was not significantly different from that of single-cropped maize. Among the three different belowground interaction treatments, partial belowground interaction in conjunction with the high maize density increased maize HI by 9% in 2014 and 42% in 2016, compared to the treatment with the treatment with no belowground interactions.

## Discussion

### Root development in relation to above- and below-ground interaction

Numerous studies have shown that intercropping increases the productivity of the whole systems (Dahmardeh et al., 2010; Yang et al., 2010; Li L. et al., 2011; Li Q. Z. et al., 2011; Oshunsanya, 2013). However, the mechanisms responsible for the yield advantages of intercropping over sole planting are poorly understood. Sharing of available soil water and nutrients between the two crops during the co-growth period has been found to contribute positively to the yield advantage (Pandey et al., 2013), while at the meantime the two crops compete for the available resources at specific growth stages (Ghosh et al., 2006; Xu et al., 2013; Zhang et al., 2016). Yield advantage is typically related to root growth and development (Xu et al., 2010; Wan et al., 2013), root distribution patterns across the rooting profile (Liu et al., 2015), and spatial variability in root traits. The root distribution patterns at the different soil depths vary with many factors, such as the morphological and physiological traits of the crops (Liu et al., 2010; Neykova et al., 2011), soil moisture (Smucker and Aiken, 1992) and nutrient contents, and plant density (Prasad and Brook, 2005). However, little information on the root characteristics of wheat-maize intercropping at different plant densities is available in the scientific literature. Working on the roots of intercropping is labor intensive and an added challenge is the difficulty of distinguishing and separating roots between the two intercrops. In the present study, we employed a modified monolith method with which the plant roots from the two intercrops were measured under the field conditions. Our results showed that the wheat-maize intercropping with no physically-inserted root barrier allowed a “complete belowground interaction” between the two intercropped species. This interspecies interaction promoted the root growth of intercropped maize significantly, reflected by the increased RWD, RLD, and root surface area density, compared with the corresponding sole cropping. Maize under a high plant density produced more roots that enhanced belowground interspecies interaction and increased crop productivity.

In interpreting the mechanisms of the yield advantages of intercropping over sole cropping in relation to root growth and distribution in the soil profile, one may consider the soil environments that affect the outcome of the belowground interspecies interactions. The growth and development of crop roots are closely related to the distribution of moisture and nutrients in the soil (Fan et al., 2016). Also, the spatial and temporal distributions of plant roots differ with crop species (Liu et al., 2011) and agronomic management practices (Vandoorne et al., 2012; Wang et al., 2012). Under dryland conditions, improving root distribution across the soil profile can enhance the crop water use efficiency (Wang et al., 2012) and thereby increasing crop productivity (Drew et al., 1973). Intercropping of crops with contrasting growth habits can improve primary resource use in both spatial and temporal contexts due to improved root distribution and connection between the species (Neykova et al., 2011). In many cases, the positive change in the soil environment brought about by one intercrop favors the growth of the other intercrop (Li et al., 2006).

### The recovery effect of maize roots

Intercropping of an earlier-sown, cool-season crop (such as spring wheat) with a later-sown, warm-season crop (such as maize) offers many advantages for the use of natural resources (Feng et al., 2010; Yin et al., 2017). However, in this planting model, the growth of the later-sown crop is often restrained due to interspecific competition for space, soil water and nutrients. When one of the intercrops is harvested, the above- and belowground parts of the remaining crop have an expanded spatial environment, which is conducive to accelerate the growth (Li et al., 2006). In the wheat/maize intercropping system, maize is at a competitive disadvantage during the early part of the co-growth period, as the maize is planted later than the wheat. However, after wheat harvest, maize accelerates its growth, which compensates for the low competitiveness occurred during the co-growth period. This phenomenon is regarded as a “recovery effect” (Li et al., 2001a). In the present study, we quantified the recovery effect by determining the differences in root growth rate between the intercropped maize and the corresponding single-cropped maize during the wheat postharvest period. We found that the intercropped maize plants in the treatment with complete belowground interaction had a significantly higher total RWD than the single-cropped maize, leading to the recovery effect of the intercropped maize. This was in contrast to the observation at the stem elongation stage when the intercropped maize had a lower RWD than the single-cropped maize due to its poor competiveness with the intercropped wheat. The “recovery effect,” reflected by the large increase of root growth traits (root length, weight, and surface area) of the intercropped maize, occurred during the wheat postharvest period when there was no longer “aboveground interspecies interaction.” Our results showed that maize was capable of overcoming any early-season competitive disadvantage brought about by intercropping it with wheat. The belowground interspecies interactions are at least partially responsible for this recovery. The significantly increased maize root growth rate (compared with the single-cropped maize) after wheat harvest was largely attributable to the increased availability of space, soil water and nutrients as there was no longer any interspecies competition for the resources. Our findings support the hypothesis by other researchers that interspecific interactions promote root growth in intercropping systems (Li L. et al., 2011). However, with the experimental design in the present study, we were unable to determine whether the maize roots actually extended into the area occupied by the wheat roots after wheat harvest. Technically, it lacked of a ready-to-use technique with which the growing maize roots can be separated from the decomposing wheat roots in the wheat strips. It was assumed that the maize plants might be able to root into the soil volume occupied by the decomposing wheat roots (Gao et al., 2010), but the assumption needs to be verified.

The outcome of the “recovery effect” can be related to many factors. Our results showed that the density of the host plant—maize impacted the magnitude of the recovery effect, and a higher maize density enhanced the recovery effect of the intercropped maize due to increased root mass stimulating belowground interspecies interaction. In a previous study, we found that plant density had a significant effect on aboveground dry matter accumulation in intercropping (Yin et al., 2017), suggesting that the belowground interspecies interactions may also stimulate the growth of the aboveground plant parts. Furthermore, the outcome of the recovery effect may be related to other soil-related factors because soil environments in the rooting zone are complex in nature and are affected by many factors, such as soil water availability (Niu et al., 2017), and soil physical (Luo et al., 2017), chemical (Grant et al., 2016), and biological (Taheri et al., 2016) properties. Agronomic practices may also affect the outcome of the recovery such as preceding crops in the rotation (Luce et al., 2016), tillage practices (Lupwayi et al., 2015), and soil microbial community structure and functionalities (Borrell et al., 2016). There is a need to elucidate those effects in the future studies to improve our understanding of the mechanisms responsible for the yield advantages of intercropping systems.

### The correlation between root-related traits and crop yields

Numerous studies have shown that intercropping systems increase crop yields significantly compared with corresponding monoculture. The increased yield is mostly attributable to increased soil water absorption and utilization (Fan et al., 2016), full use of belowground space (Agele, 2010; Devi et al., 2014), and improved rate of nutrient utilization (Levangbrilz and Biondini, 2003). Also, studies have revealed that root distribution affects crop growth and final yield (Li et al., 2001b; Kashiwagi et al., 2006; Gao et al., 2010; Shiponeni et al., 2014). An overlapping of roots occurs in a two-crop intercropping system (Li et al., 2006) which leads to greater root mass and higher crop yields (Yang et al., 2010). The enhanced root growth and development partially offsets the interspecific competitions for light (Amossé et al., 2013), heat (Knörzer et al., 2011), carbon dioxide (Shili Touzi et al., 2010), and other resources (Amossé et al., 2013; Dolijanović et al., 2013). A significant addition to the known features of intercropping systems from the present study is that belowground interspecies interactions, stimulated through the root growth and development of the intercrops, are partially responsible for the increased crop yield. Ecological principles indicate that competition and facilitation coexist simultaneously in a co-growing plant community. Our results showed that the belowground interspecies interaction helped overcome the competitive disadvantage of the maize encountered in the early co-growth period. In a previous study, we found that maize had a “super-compensation effect” for increasing the yield of the wheat/maize intercropping system but the mechanism was undetermined (Yin et al., 2017). Now, we understand that the “super-compensation effect” is partly attributable to the enhanced belowground interspecies interactions that help alleviate aboveground inter-plant competition.

## Conclusion

Belowground interspecies interaction promoted the root growth and development as reflected by the increased root traits - RWD, RLD, and RSAD in the intercropped maize. During the co-growth period, the intercropped wheat had greater RWD, RLD, and RSAD than the single-cropped wheat across the 20–100 cm rooting zone. The intercropped maize had a lower RWD, RLD, and RSAD than single-cropped maize. At wheat postharvest, the intercropped maize increased RWD, RLD, and RSAD rapidly, enabling the “recovery effect” of the impaired maize growth experienced during the co-growth period, and the higher maize plant density enhanced the recovery effect. Comparisons for the differences in root weight between intercropped maize and single-cropped maize gave a quantitative assessment of the recovery effect: the belowground interspecies interaction contributed 143% to the recovery effect, the CRO contributed 35%, and the CWN contributed 80%. A complete belowground interaction is the key to promote the recovery effect, and a high maize density enhances the recovery effect.

## Author contributions

QC and YW conceived and designed the experiment; YW, QC, and FF performed the statistical analyses; YW, YQ, AY, and CZ were involved in field data collection. All authors contributed to writing the manuscript.

### Conflict of interest statement

The authors declare that the research was conducted in the absence of any commercial or financial relationships that could be construed as a potential conflict of interest.

## References

[B1] Agegnehu G. Ghizaw A. Sinebo W. (2008). Yield potential and land-use efficiency of wheat and faba bean mixed intercropping. Agron. Sustain. Dev. 28, 257–263. 10.1051/agro:2008012

[B2] Agele S. (2010). Dynamics of soil properties, soil fauna activity, pepper nutrition and fruit yield under plantain based intercropping system in a humid rainforest environment. Arch. Agron. Soil Sci. 56, 265–283. 10.1080/03650340903092200

[B3] Ahmad G. Mehdi D. Barat A. S. Mahmoud R. (2010). Effect of maize (*Zea mays* L.)-Cowpea (*Vigna unguiculata* L.) intercropping on light distribution, soil temperature and soil moisture in arid environment. J. Food Agric. Environ. 8, 102–108. Available online at: https://www.researchgate.net/publication/268411733_Effect_of_maize_Zea_mays_L_-_Cowpea_Vigna_unguiculata_L_intercropping_on_light_distribution_soil_temperature_and_soil_moisture_in_arid_environment

[B4] Amossé C. Jeuffroy M. H. David C. (2013). Relay intercropping of legume cover crops in organic winter wheat: effects on performance and resource availability. Field Crops Res. 145, 78–87. 10.1016/j.fcr.2013.02.010

[B5] Beaver R. J. Melgar M. (1999). Analysis of yield-density models for intercropping experiments. Biometrical J. 41, 995–1011. 10.1002/(SICI)1521-4036(199912)41:8<995::AID-BIMJ995>3.0.CO;2-H

[B6] Borrell A. N. Shi Y. Gan Y. Bainard L. D. Germida J. J. Hamel C. (2016). Fungal diversity associated with pulses and its influence on the subsequent wheat crop in the Canadian prairies. Plant Soil 414, 13–31. 10.1007/s11104-016-3075-y

[B7] Chai Q. Qin A. Z. Gan Y. T. Yu A. Z. (2014). Higher yield and lower carbon emission by intercropping maize with rape, pea, and wheat in arid irrigation areas. Agron. Sustain. Dev. 34, 535–543. 10.1007/s13593-013-0161-x

[B8] Dahmardeh M. Ghanbari A. Syahsar B. A. Ramrodi M. (2010). The role of intercropping maize (*Zea mays* L.) and cowpea (*Vigna unguiculata* L.) on yield and soil chemical properties. Afr. J. Agr. Res. 5, 631–636. 10.5897/AJAR09.607

[B9] Devi K. N. Shamurailatpam D. Singh T. B. Athokpam H. S. Singh N. B. Singh N. G. (2014). Performance of lentil (*Lens culinaris* M.) and mustard (*Brassica juncea* L.) intercropping under rainfed conditions. Aust. J. Crop Sci. 8, 284–289. Available online at: https://www.researchgate.net/publication/259475493_Performance_of_lentil_Lens_culinaris_M_and_mustard_Brassica_juncea_L_intercropping_under_rainfed_conditions

[B10] Dolijanović Z. Oljača S. Kovacević D. Simić M. Momirović N. Jovanović Z. (2013). Dependence of the productivity of maize and soybean intercropping systems on hybrid type and plant arrangement pattern. Genet. Belgr. 45, 135–144. 10.2298/GENSR1301135D

[B11] Drew M. C. Saker L. R. Ashley T. W. (1973). Nutrient supply and the growth of the seminal root system in barley I. The effect of nitrate concentration on the growth of axes and laterals. J. Exp. Bot. 24, 1189–1202. 10.1093/jxb/24.6.1189

[B12] Fan J. Mcconkey B. Wang H. Janzen H. (2016). Root distribution by depth for temperate agricultural crops. Field Crops Res. 189, 68–74. 10.1016/j.fcr.2016.02.013

[B13] Fan Z. L. Chai Q. Huang G. B. Yu A. Z. Huang P. Yang C. H. (2013). Yield and water consumption characteristics of wheat/maize intercropping with reduced tillage in an Oasis region. Eur. J. Agron. 45, 52–58. 10.1016/j.eja.2012.10.010

[B14] Feng F. X. Huang G. B. Chai Q. Yu A. Z. (2010). Tillage and straw management impacts on soil properties, root growth, and grain yield of winter wheat in Northwestern China. Crop Sci. 50, 1465–1473. 10.2135/cropsci2008.10.0590

[B15] Gan Y. T. Siddique K. H. M. Turner N. C. Li X. G. Niu J. Y. Yang C. (2013). Chapter seven–ridge-furrow mulching systems-an innovative technique for boosting crop productivity in semiarid rain-fed environments. Adv. Agron. 118, 429–476. 10.1016/B978-0-12-405942-9.00007-4

[B16] Gao Y. Duan A. W. Qiu X. Q. Liu Z. G. Sun J. S. Zhang J. P. (2010). Distribution of roots and root length density in a maize/soybean strip intercropping system. Agr. Water Manage. 98, 199–212. 10.1016/j.agwat.2010.08.021

[B17] Ghosh P. K. Mohanty M. Bandyopadhyay K. K. Painuli D. K. Misra A. K. (2006). Growth, competition, yields advantage and economics in soybean/pigeonpea intercropping system in semi-arid tropics of India: II. Effect of nutrient management. Field Crops Res. 96, 90–97. 10.1016/j.fcr.2005.05.010

[B18] Grant C. A. O'Donovan J. T. Blackshaw R. E. Harker K. N. Johnson E. N. Gan Y. T. (2016). Residual effects of preceding crops and nitrogen fertilizer on yield and crop and soil N dynamics of spring wheat and canola in varying environments on the Canadian prairies. Field Crops Res. 192, 86–102. 10.1016/j.fcr.2016.04.019

[B19] Hu F. L. Chai Q. Yu A. Z. Yin W. Cui H. G. Gan Y. T. (2014). Less carbon emissions of wheat–maize intercropping under reduced tillage in arid areas. Agron. Sustain. Dev. 35, 701–711. 10.1007/s13593-014-0257-y

[B20] Hu F. L. Gan Y. T. Cui H. Y. Zhao C. Feng F. X. Yin W. (2016). Intercropping maize and wheat with conservation agriculture principles improves water harvesting and reduces carbon emissions in dry areas. Eur. J. Agron. 74, 9–17. 10.1016/j.eja.2015.11.019

[B21] Hu X. T. Chen H. Wang J. Meng X. B. Chen F. H. (2009). Effects of soil water content on cotton root growth and distribution under mulched drip irrigation. Agr. Sci. China 8, 709–716. 10.1016/S1671-2927(08)60269-2

[B22] Kashiwagi J. Krishnamurthy L. Crouch J. H. Serraj R. (2006). Variability of root length density and its contributions to seed yield in chickpea (*Cicer arietinum* L.) under terminal drought stress. Field Crop Res. 95, 171–181. 10.1016/j.fcr.2005.02.012

[B23] Knörzer H. Grözinger H. Graeff-Hönninger S. Hartung K. Piepho H. P. Claupein W. (2011). Integrating a simple shading algorithm into CERES-wheat and CERES-maize with particular regard to a changing microclimate within a relay-intercropping system. Field Crops Res. 121, 274–285. 10.1016/j.fcr.2010.12.016

[B24] Landhäusser S. M. Lieffers V. J. Mulak T. (2006). Effects of soil temperature and time of decapitation on sucker initiation of intact Populus tremuloides root systems. Scand. J. Forest Res. 21, 299–305. 10.1080/02827580600813313

[B25] Levangbrilz N. Biondini M. E. (2003). Growth rate, root development and nutrient uptake of 55 plant species from the Great Plains Grasslands, USA. Plant Ecol. 165, 117–144. 10.1023/A:1021469210691

[B26] Li L. Sun J. H. Zhang F. S. (2011). Intercropping with wheat leads to greater root weight density and larger below-ground space of irrigated maize at late growth stages. Soil Sci. Plant Nutr. 57, 61–67. 10.1080/00380768.2010.548307

[B27] Li L. Sun J. H. Zhang F. S. Li X. L. Rengel Z. Yang S. C. (2001a). Wheat/maize or wheat/soybean strip intercropping. II. Recovery or compensation of maize and soybean after wheat harvesting. Field Crops Res. 71, 173–181. 10.1016/S0378-4290(01)00157-5

[B28] Li L. Sun J. H. Zhang F. S. Li X. L. Yang S. C. Rengel Z. (2001b). Wheat/maize or wheat/soybean strip intercropping : I. Yield advantage and interspecific interactions on nutrients. Field Crops Res. 71, 123–137. 10.1016/S0378-4290(01)00156-3

[B29] Li L. Sun J. Zhang F. Guo T. Bao X. Smith F. A.. (2006). Root distribution and interactions between intercropped species. Oecologia 147, 280–290. 10.1007/s00442-005-0256-4 16211394

[B30] Li Q. Z. Sun J. H. Wei X. J. Christie P. Zhang F. S. Li L. (2011). Overyielding and interspecific interactions mediated by nitrogen fertilization in strip intercropping of maize with faba bean, wheat and barley. Plant Soil 339, 147–161. 10.1007/s11104-010-0561-5

[B31] Liu L. Gan Y. Bueckert R. Van Rees K. Warkentin T. (2010). Fine root distributions in oilseed and pulse crops. Crop Sci. 50, 222–226. 10.2135/cropsci2009.03.0156

[B32] Liu L. Gan Y. T. Bueckert R. Van Rees K. (2011). Rooting systems of oilseed and pulse crops I: temporal growth patterns across the plant developmental periods. Field Crops Res. 122, 256–263. 10.1016/j.fcr.2011.04.002

[B33] Liu Y. X. Zhang W. P. Sun J. H. Li X. F. Christie P. Li L. (2015). High morphological and physiological plasticity of wheat roots is conducive to higher competitive ability of wheat than maize in intercropping systems. Plant Soil 397, 387–399. 10.1007/s11104-015-2654-7

[B34] Luce M. S. Grant C. A. Ziadi N. Zebarth B. J. O'donovan J. T. Blackshaw R. E. (2016). Preceding crops and nitrogen fertilization influence soil nitrogen cycling in no-till canola and wheat cropping systems. Field Crops Res. 191, 20–32. 10.1016/j.fcr.2016.02.014

[B35] Luo Z. Gan Y. Niu Y. Zhang R. Li L. Cai L. (2017). Soil quality indicators and crop yield under long-term tillage systems. Exp. Agric. 53, 497–511. 10.1017/S0014479716000521

[B36] Lupwayi N. Z. Harker K. N. O'donovan J. T. Turkington T. K. Blackshaw R. E. Hall L. M. (2015). Relating soil microbial properties to yields of no-till canola on the Canadian prairies. Eur. J. Agron. 62, 110–119. 10.1016/j.eja.2014.10.004

[B37] Muoneke C. O. Ogwuche M. A. O. Kalu B. A. (2007). Effect of maize planting density on the performance of maize/soybean intercropping system in a guinea savannah agroecosystem. Afr. J. Agr. Res. 2, 667–677. Available online at: https://www.researchgate.net/publication/228667314_Effect_of_maize_planting_density_on_the_performance_of_maizesoybean_intercropping_system_in_a_guinea_savannah_agroecosystem

[B38] Neykova N. Obando J. Schneider R. Shisanya C. Thiele B. S. Thomas F. M. (2011). Vertical root distribution in single-crop and intercropping agricultural systems in Central Kenya. J. Plant Nutr. Soil Sci. 174, 742–749. 10.1002/jpln.201000314

[B39] Niu Y. N. Bainard L. Bandara M. S. Hamel C. Gan Y. T. (2017). Soil residual water and nutrients explain about 30% of the rotational effect in 4-year pulse-intensified rotation systems. Can. J. Plant Sci. 97, 852–864. 10.1139/CJPS-2016-0282

[B40] Ong C. K. (1995). The “dark side” of Intercropping: manipulation of soil resources,” in Ecophysiology of Tropical Intercropping, eds Sinoquet H. Cruz P. (Paris: INPR), 45–66.

[B41] Oshunsanya S. O. (2013). Spacing effects of vetiver grass (Vetiveria nigritana Stapf) hedgerows on soil accumulation and yields of maize-cassava intercropping system in Southwest Nigeria. Catena 104, 120–126. 10.1016/j.catena.2012.10.019

[B42] Pandey I. B. Singh S. K. Tiwari S. (2013). Integrated nutrient management for sustaining the productivity of pigeonpea (*Cajanus cajan*) based intercropping systems under rainfed condition. Indian J. Agron. 58, 192–197. Available online at: https://www.researchgate.net/publication/289573564_Integrated_nutrient_management_for_sustaining_the_productivity_of_pigeonpea_Cajanus_cajan_based_intercropping_systems_under_rainfed_condition

[B43] Prasad R. B. Brook R. M. (2005). Effect of varying maize densities on intercropped maize and soybean in Nepal. Exp. Agr. 41, 365–382. 10.1017/S0014479705002693

[B44] Qin A. Z. Huang G. B. Chai Q. Yu A. Z. Huang P. (2013). Grain yield and soil respiratory response to intercropping systems on arid land. Field Crop Res. 144, 1–10. 10.1016/j.fcr.2012.12.005

[B45] Romero P. Gil-Muñoz R. Del Amor F. M. Valdés E. Fernández J. I. Martinez-Cutillas A. (2013). Regulated deficit irrigation based upon optimum water status improves phenolic composition in monastrell grapes and wines. Agr. Water Manage. 121, 85–101. 10.1016/j.agwat.2013.01.007

[B46] Schenk H. J. (2006). Root competition: beyond resource depletion. J. Ecol. 94, 725–739. 10.1111/j.1365-2745.2006.01124.x

[B47] Shili Touzi I. De Tourdonnet S. Launay M. Dore T. (2010). Does intercropping winter wheat (*Triticum aestivum*) with red fescue (*Festuca rubra*) as a cover crop improve agronomic and environmental performance? A modeling approach. Field Crops Res. 116, 218–229. 10.1016/j.fcr.2009.11.007

[B48] Shiponeni N. N. Carrick P. J. Allsopp N. Hoffman M. T. Ward D. (2014). Effects of root competition and soils on seedling establishment at the ecotone between an arid grassland and succulent shrubland in South Africa. J. Veg. Sci. 25, 402–410. 10.1111/jvs.12082

[B49] Smit A. L. Bengough A. G. Engels C. Noordwijk M. V. Pellerin S. Geijn S. C. (2000). Root Methods a Hand Book. Heidelberg: Springer.

[B50] Smucker A. J. M. Aiken R. M. (1992). Dynamic root responses to water deficits. Soil Sci. 154, 281–289. 10.1097/00010694-199210000-00004

[B51] Taheri A. E. Hamel C. Gan Y. T. (2016). Cropping practices impact fungal endophytes and pathogens in durum wheat roots. Appl. Soil Ecol. 100, 104–111. 10.1016/j.apsoil.2015.12.007

[B52] Vandermeer J. H. (1989). The Ecology of Intercropping. Cambridge: Cambridge University Press.

[B53] Vandoorne B. Mathieu A. S. Van Den Ende W. Vergauwen R. Périlleux C. Javaux M.. (2012). Water stress drastically reduces root growth and inulin yield in *Cichorium intybus* (var. sativum) independently of photosynthesis. J. Exp. Bot. 63, 4359–4373. 10.1093/jxb/ers095 22577185 PMC3421980

[B54] Wan Y. Yan Y. H. Yang T. W. Liu W. G. Wang X. C. (2013). Responses of root growth and nitrogen transfer metabolism to uniconazole, a growth retardant, during the seedling stage of soybean under relay strip intercropping system. Commun. Soil Sci. Plant 44, 3267–3280. 10.1080/00103624.2013.840838

[B55] Wang H. Inukai Y. Yamauchi A. (2006). Root development and nutrient uptake. Crit Rev in Plant Sci. 25, 279–301. 10.1080/07352680600709917

[B56] Wang X. Gan Y. Hamel C. Lemke R. Mcdonald C. (2012). Water use profiles across the rooting zones of various pulse crops. Field Crops Res. 134, 130–137. 10.1016/j.fcr.2012.06.002

[B57] Wilson J. B. (1988). Shoot competition and root competition. J. Appl. Ecol. 25, 279–296. 10.2307/2403626

[B58] Xu B. Li F. Shan L. (2010). Seasonal root biomass and distribution of switchgrass and milk vetch intercropping under 2:1 row replacement in a semiarid region in Northwest China. Commun. Soil Sci. Plant 41, 1959–1973. 10.1080/00103624.2010.495806

[B59] Xu H. Bi H. Gao L. Yun L. Chang Y. Xi W. (2013). Distribution and morphological variation of fine root in a walnut-soybean intercropping system in the loess plateau of china. Int. J. Agric. Biol. 15, 998–1002. Available online at: https://www.researchgate.net/publication/288456398_Distribution_and_morphological_variation_of_fine_root_in_a_walnut-soybean_intercropping_system_in_the_loess_plateau_of_china

[B60] Yang C. H. Chai Q. Huang G. B. (2010). Root distribution and yield responses of wheat/maize intercropping to alternate irrigation in the arid areas of northwest China. Plant Soil Environ. 56, 253–262. 10.17221/251/2009-PSE

[B61] Yin W. Chen G. P. Feng F. X. Guo Y. Hu F. L. Chen G. D. (2017). Straw retention combined with plastic mulching improves compensation of intercropped maize in arid environment. Field Crop Res. 204, 42–51. 10.1016/j.fcr.2017.01.005

[B62] Yin W. Yu A. Z. Chai Q. Hu F. L. Feng F. X. Gan Y. T. (2015). Wheat and maize relay-planting with straw covering increases water use efficiency up to 46%. Agron. Sustain. Dev. 35, 815–825. 10.1007/s13593-015-0286-1

[B63] Zhang F. S. Li L. (2003). Using competitive and facilitative interactions in intercropping systems enhances crop productivity and nutrient-use efficiency. Plant Soil 248, 305–312. 10.1023/A:1022352229863

[B64] Zhang W. P. Liu G. C. Sun J. H. Fornara D. Zhang L. Z. Zhang F. F. (2016). Temporal dynamics of nutrient uptake by neighbouring plant species: Evidence from intercropping. Funct. Ecol. 31, 469–479. 10.1111/1365-2435.12732

